# Design and automation at the end of modernity: the teachings of the pandemic

**DOI:** 10.1007/s44223-022-00001-0

**Published:** 2022-06-25

**Authors:** Mario Carpo

**Affiliations:** University College London, London, UK

## Abstract

Many in the design community have long claimed that digital mass-customization is cheaper, faster, smarter and more environmentally sustainable than the mechanical mass-production of standardized industrial products; and that the electronic transmission of information is cheaper, faster, smarter, and more environmentally sustainable than the mechanical transportation of people and goods. The global pandemic has tragically proven that a computational alternative to the modern, mechanical way of making, working, and living, now exists, and it is viable. When we had to shut down corporate offices, global megafactories, suburban shopping malls, and intercontinental airports, we did. We did because we had to; but also because today's technology already allows us to do so.

The medium is the message. That was originally the title of a chapter in a ponderous scholarly book by Marshall McLuhan, *Understanding Media*, first published in 1964, where McLuhan famously elaborated on his theory of a new "global village" of electronic communication brought about by radio and television. McLuhan soon realized that he could best explain TV by being on TV himself, and he did so very well for many years. But let's take McLuhan statement at face value, and try to apply it outside of its original context. What's our medium today? Or, rather, what has it been for the last 2 years? I am a teacher; and you may easily guess--the main tool of my trade, for the last two years, has been Zoom. So, if that is the medium, what is the message? There is an immediate message, evidently: When we are on Zoom we *are* meeting, somehow, but we are not really together: we are not at school. My home university, for example, was almost entirely closed for 18 months, and after a number of partial reopenings, many lectures and meetings are still online as I write (April 2022).

Which suggests that, alongside the immediate message I mentioned, there may be a more general but subliminal message at play here. As we know, the global travel and transportation infrastructure of modern industrial world all of a sudden came to a grinding halt in March 2020, when it just shut down--entirely for some months, and then restarting partially, and intermittently, following the ebb and flow of the pandemic. With travel and transportation suspended, the entire technical logic of the modern, industrial, machine-made environment simply imploded. This is what many today call the Anthropocene, a term often invoked to designate the modern mechanical world issued from the Industrial Revolution and based on standardized mass-production, global mechanical transportation, and the unlimited burning of fossil fuels. What we have seen for the last two years is simply, the collapse of *that* world--the collapse of the Anthropocene. We all thought that the Anthropocene would come to an end, little by little, and over time, due to global warming and climate change. Instead, the end of the Anthropocene came not by way of global warming, but of global infection; not due to climate change, but due to viral change--and it came suddenly, the space of a fortnight, in March 2020.

The speed and timing and nature of the catastrophe were of course unprecedented. Yet for us, in the design professions, the end of the Anthropocene by itself should not have come as a surprise. After all, we have long been claiming that the technical logic of industrial modernity is obsolete, and due to be replaced by the technical logic of post-industrial electronic computation. In particular, we have long been claiming that the electronic transmission of information is faster, cheaper, smarter, and more environmentally sustainable than the mechanical transportation of people and goods. And likewise, we have long been claiming that the distributed, automated, and digitally mass-customized fabrication of physical goods is faster, cheaper, smarter, and more environmentally sustainable than standardized industrial mass production. The global catastrophe of the pandemic has tragically vindicated many of these long-held assumptions.

Let's take a practical example: to keep to our own daily experience for the last two years, let's look at the way the pandemic has changed higher education. You will not be surprised to learn that one weekend in March 2020, without forewarning, and all of a sudden, I had to transfer all my classes, lectures, tutorials, seminars from real classes to virtual ones, while trying to figure out what we can and cannot do in this new electronic format. As all the media we use inevitably feed-back on the kind of messages we can convey, this is, as we all know at this point, a complicated matter. For me it was a steep learning curve, because I had no experience of remote teaching, I had never done it before, so I had plenty of catching up to do--technically and intellectually.

But this is where I had to stop, and ask myself: why on earth did I not at least try to start and practice some remote teaching a bit earlier; why did I wait so long--why did I not start long ago? All the technology we use today to teach remotely has been widely available (at least on campuses) since the mid 1990s. And many bold and daring experiments of e-learning and virtual classes were tested and tried back then, in the 1990s, when the technology was new and promising and exciting, and many thought it could change teaching forever, and for the better. Mark Taylor, the Derrida scholar, a professor of philosophy and religion now at Columbia University, started teaching online classes at Williams College in the early 1990s, and his first experiments are reported and discussed in a book he published in 1994.^1^ And yet, in spite of such an early and promising start, e-teaching never really caught on. Even in more recent times, the rise of MOOCS (Massive Online Open Courses) has been widely dismissed, and it has been disparaged by the global academic establishment, as well as seen with suspicion by activists of all ilk.

But then if we stop again, step back, and have a look at the bigger picture, it becomes evident that this pattern of rise and fall in the appeal of digital technologies--this sequence in two steps: first, a period of great technological optimism in the 1990s; followed by a tide of deep distrust of all things digital as of the early 2000s--is part to a larger, more general trend. And this is a trend that we, in the design professions, are quite familiar with, because this is a story partly at least of our own making. For better or worse, starting from the early 1990s, we have been among the inventors, the protagonists, the pioneers of the digital turn. We embraced and adopted digital tools sooner than many other trades and professions, and in the process we came up with ideas that have shaped our general understanding of digital technologies and of digital culture at large.

The story of the invention of the digital turn in architecture in the 1990s is well known. Back then, in the 1990s, designers and architects were the first to understand the revolutionary potential of seamless CAD-CAM integration. That new technical logic promised the mass-production of variations at no extra-cost, and some of us around that time came up with a new conceptual framework to define this new technical object, and to come to terms with this new way of making--which we now call digital mass customization. At the same time, new parametric software for the design of complex geometrical shapes and smooth, streamlined surfaces in particular, created a new generation of architectural objects, which around that time Greg Lynn started calling Blobs. Blobs have since taken over the world, and they are still thriving. They are not called Blobs any more, but that irrelevant because blobs come and go--after all, that's just an architectural style--whereas the real revolution was, and still is, in the technical logic of digital design and fabrication underpinning them. Regardless of form and style, this was a technical revolution with the potential the change the way we design and make, produce and consume, buy and sell, almost everything.

Digital mass customization promises a new mode of design and fabrication that is unaffected by economies of scale: a new mode of production where making one, or one million identical replications of the same item will come at the same unit cost; where making one million items all the same, or making all of them all different, will cost the same. This is, simply, the reversal of all the principles of industrial mass production, and of the economics of modern industrial modernity. Architects and designers in the 1990s where the first to intuit this new technical logic, and to come up with a theory to describe and explain it. And if this were not enough, parallel to this story--which is all about CAD-CAM and digital tectonics: notation and fabrication--there was another story unfolding at the same time, deeply intertwined with it, and often told by the same protagonists, in the same schools and venues.

In 1995 Bill Mitchell, then the Dean of Architecture and Planning at the MIT, published a seminal little book, *City of Bits*, where he argued that many things we do--economic activities and social activities alike--would soon migrate from physical space to a new electronic space, called the internet; and as a result, many building types that defined the modern city would soon be made obsolete. Mitchell made a long list of functions and buildings slated to disappear: local bank branches, because electronic banking would replace physical paperwork and face-to-face transactions; travel agencies, because travelers would buy tickets from airlines websites; newspaper kiosks, because we would read newspapers online; post offices, because we would use e-mail; record stores and bookstores, because we would buy records and books online, or download them as digital files; libraries, because we would check catalogues and soon read digitized books remotely rather than on site; and last but not least office buildings, because at some point thanks to the internet we would all work from home, without having to commute to an office.

The internet: that was the place where all these functions--meaning, most of our life--was soon meant to be taking place. Remember, that was 1995, when many of us didn't even have an internet access. The internet itself was not new in 1995, as it derived from an older military network called the ARPANET. But in 1989 and 1990 Tim Berner Lee, then working in Geneva, invented the http, the html, hence the World Wide Web, which gave every user of the internet the possibility to "surf the web", navigating the entire internet from one hyperlink to the next, without having to type in a new internet address for each connection to a different server. With the WWW the internet suddenly became a user-friendly space, ready for popular use, and primed for commercial exploitation. Many started to think that the internet would be the future of retail: Amazon sold its first book in the summer of 1995. And the company was not called Amazon; it was called, as it still is, Amazon.com. From the start, the name of the company was its internet address, or more precisely its URL--its address on the WWW.

From brick and mortar to virtual reality: as one frequently cited proverb of the time went, for every megabyte of memory you install on your hard disk, one square foot of retail space downtown will disappear.^2^ Strange as it may seem today, back then everyone thought that was a splendid idea. And around that time some also started to point out that a reduction in the mechanical transportation of persons and goods could be environmentally friendly (or, as we would say today, would reduce our "carbon footprint"). Bill Mitchell did not stop there: as an architect and urban theorist, he pointed out that the rise of the internet meant, simply, the end of the modernist city, based on the separation of functions and their concentration in specialized "zones": parts of the city where we live but we do not work; other parts where we work but do not live, and parts we shop or go to the movies or have fun but we neither live or work, while permanently commuting from one place to the next. Modernist zoning, Mitchell argued, was over: with the shift from mechanical transportation to electronic transmission many of these activities could be transacted anywhere--because they would mostly take place on the internet. Hence the functional segregation of modernist cities would stop, and cities could go back to being integrated, organic, multi-functional, multi-purposes, and messy--as they had always been before the rise of modern urbanism. At the same time many of these dispersed, distributed, despatialized activities would merge to consolidate in a new, reconfigured, electronically connected physical place: the dwelling; the new domestic space; home. No longer *at home* but *@home* (Fig. 1).

**Fig. 1:**
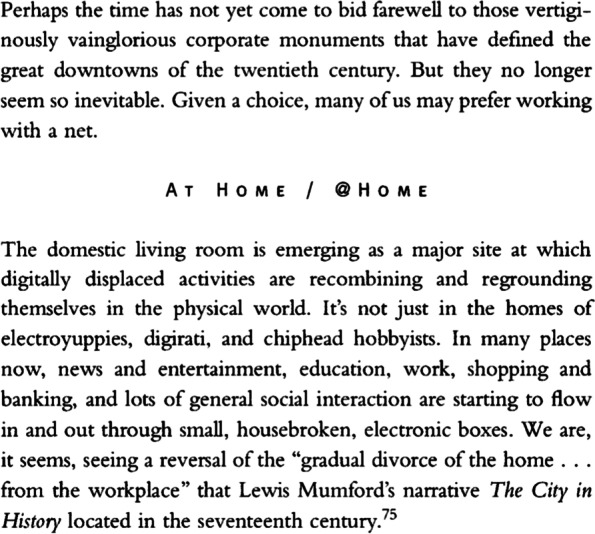
William J. Mitchell, *City of Bits. Space, Place, and the Infobahn* (Cambridge, MA: MIT Press, 1995), 98

That was 1995. After working from home non-stop for most of the last two years we can certainly see some of the downsides of teleworking. Yet, 25 years ago, everyone thought that despatialization, namely the migration of human activities from physical space to the "cyberspace" (a popular term at the time)^3^ was the future--and that future, seen from 1995, looked good. Nor were designers the only ones to jump into cyberspace. In the late 90s the digital revolution was widely seen as the final fix for almost every problem created by the industrial revolution: it was a general leap of faith, a sudden surge of unrestrained enthusiasm for the redemptive power of the internet, and of digital technologies in general. If he had lived to see it, Karl Marx would have said that the digital age was the inevitable "overcoming" of the industrial age.

Of course nobody was reading Karl Marx in 1995. Instead, everyone was buying internet companies on the stock exchange. That was the beginning of what we now call the Internet Bubble. The valuation of all dot-com companies (companies with a dot and a "com" in their name, implying that they were doing business on the internet, or just suggesting that they would do so at some point) soared. Between January 1995 and March 2000 the NASDAQ composite index, where many of these young companies were quoted, rose by almost 600 per cent. As the then chairman of the Federal Reserve of the US, Alan Greenspan, famously said, that was not due to "irrational exuberance": valuations were soaring because the internet made our work in general more productive, and many things easier to find, buy, or sell, hence cheaper. Thanks to the internet, we were told, we were all doing *more with less*: more work, more reading, more teaching, more learning, more researching, more interacting, more dating--you name it. This newfound riches, in the years after the fall of the Berlin wall, were often called the dividends of peace. But the meteoric rise of the valuation of all dot-com companies of the time could equally have been called *the dividends of despatialization*: cyberspace costs less than physical space, and it works better. Therefore, when the economy, in general, moves from physical space to cyberspace--to the internet--we all become wealthier.

That seemed too good to be true--until it wasn't. The NASDAQ peaked on March 10, 2000. It lost 80 per cent of its value in the 18 months that followed. That was the dot-com crash, aka the burst of the Internet Bubble. Many tech companies disappeared; Amazon itself, for example, barely survived, after losing 88 per cent of its market capitalization. The NASDAQ took 15 years to crawl back to its value of March 2000. Stock exchange crashes tend to coincide with deep and sudden changes in culture and in society at large; the dot-com crash was no exception. As the NASDAQ plunged, in 2000 and 2001, the general mood shifted, suddenly, from techno-friendly to technophobic. In the contrite climate of those post-crash years (which were also the post 9/11 years) few still saw the internet as a benevolent, or even as promising technology.

The anti-internet backlash was swift, and predictable. As many had warned from the start, technology should not replace human contact; there can be no community without physical proximity. For Christian phenomenologists, always overrepresented in the design professions, the elision of human dialogue started with the invention of alphabetic writing: if we write, we use a technology--the alphabet--to transmit our voice to others in the absence of our body. That was the original sin of all media technologies: after that, things could only get from bad to worse; the internet was just more of the same. And the backlash continued, in a sense to this day: a few years into the new millennium the so-called social media reinvented the internet; in recent times we have learned to fear their intrusion on our privacy. Furthermore, by abolishing all traditional tools for thoughtful moderation, and giving unmediated voice to so many dissenters, outliers, and misfits, the internet is seen by many as the primary technical cause of the rise of populism. That may as well be true, although I suspect that if I had been a Roman Catholic cleric around 1540 I would have said the same of the use of the new barbaric technology of print by the likes of John Calvin or Martin Luther.

I drafted a first, shorter version of this paper in the spring of 2020, during the first British lockdown, while self-isolating in my London apartment, like hundreds of millions of Europeans, contemplating the unfolding of an unspeakable catastrophe.^4^ From my living room windows I used to see, in normal times, the distant flow of airplanes gliding into Heathrow, evenly spaced, three minutes from one another. For most of 2020 and 2021 I could only see a handful--and most of them carrying cargo, not passengers. Only a few months before the pandemic Greta Thunberg still incited us, by words and deeds, to "flight shaming"--meaning, to "shame" those traveling by plane, due to carbon footprint of air travel; she won her battle big way, although not for the right reasons. As the carbon-heavy economy of the industrial age collapsed in 2020--and in most of the world has not yet gone back to its prepandemic levels-- we may have already significantly delayed the timeline of global warming. Only a few months before the pandemic some climate activists were more or less openly advocating the elimination of part of the human population as the only way to save the planet: well, there you go.

Meanwhile, something we have already learned from this catastrophe is that real viruses can be more lethal than internet viruses. The coronavirus did not spread on the internet: it traveled by plane, boat, and rail. The coronavirus was born and bred as a pure product of the industrial mechanical age. If a few months back, when this all started, we had already been using more internet, and flying less (as we are doing now, by necessity not by choice), many lives would have been saved, because the virus would have had fewer conduits for spreading. So perhaps, in retrospect, this is exactly what we should have been doing--what we should have started doing long ago. Schools, stores, and factories have now reopened, somehow--but many offices have not, for example, and many of us are still working from home, and often shopping from home. This is happening because the pandemic has shown that the traditional way of working--the "anthropocenic" way of working, based on presence in the flesh, travel and transportation--is no longer our only option. When the pandemic obliged us to work from home, we did. We did because we had to, but also because today's technology allows us to do so. During the pandemic we have learnt that today's electronic communications can already effectively replace plenty of face time, thus making plenty of human travel unnecessary. The alternative to air travel is not sailing boat travel. It's Zoom.

Blue-collar work cannot yet be despatialized as effectively as white-collar work, but that's not too far away in the future either: fully automated robotic fabrication is already current in some industries (and in design schools we are actually quite good at it). Robotic factories are mostly immune to economies of scale: they do not need to scale up to break even, hence they can be smaller and located closer to their markets, thus reducing the global transportation of raw materials and of mass-produced goods and components. Anecdotally, but meaningfully, right at the start of the pandemic some among my friends and colleagues, like Manuel Jimenez Garcia at the Bartlett, or Jenny Sabin at Cornell, started to convert their 3D printers and robotic arms to produce PPE protective equipment for medics and hospital workers--on site, on specs and on demand. Because this is indeed the point; this is what robotic fabrication was always meant to do. The same robotic arm that made a Zaha Hadid table set last week can make face shields for medical staff today, only a few miles from a hospital in need: no airport needed for delivery. Once again, the emergency of the pandemic has proven a long-held assumption of digital design theory: the next wave of robotic fabrication will not automate the industrial factory--that's already done, and we shall need less and less of that. The new generation of robotic fabrication will *eliminate* the industrial factory, and replace it with a network of smarter, nimbler, reprogrammable micro-factories: flexible installations that will make things only when needed, where needed, as needed; using local material, locally produced energy, and a global electronic network of data and computing power. The next wave of automation will not replace industrial workers: it will reinvent the preindustrial artisan--and its inherently sustainable circular economy (Fig. 2).

**Fig. 2:**
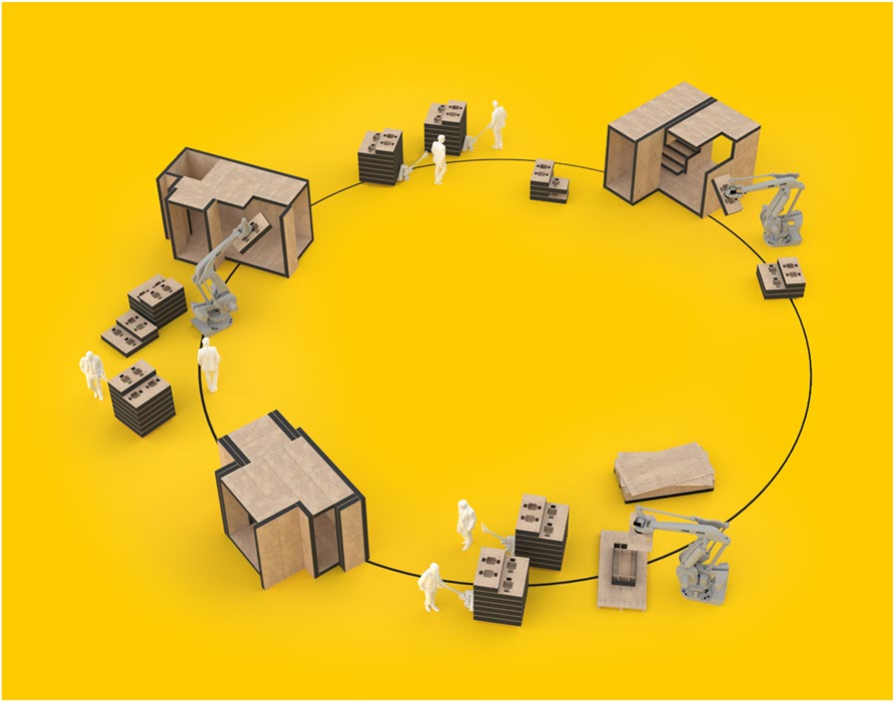
Automated Architecture (AUAR) develops a network of robotic microfactories for affordable housing. Courtesy AUAR, Gilles Retsin

Now I know full well--and I am the first to admit, and to regret--that many things have gone wrong in the way we approach computational tools, and even digital culture in general, in the last thirty years. Digital theory has been infiltrated by a number of malevolent ideologies--and I do not only mean right-wing libertarianism, the political ideology of choice for many venture capitalists that invested in, promoted, and profited from, digital technologies. That's the easiest target but it's only the tip of the iceberg. More generally speaking, and in my opinion more dangerously, the ideology of the so-called post-modern science of complexity has by now deeply pervaded contemporary computer science, offering a conceptual framework driven by the belief in the supernatural power of emergence and self-organization; by the search for the irrational leap in the dark, for the drastic and sudden reset, for the creative quantum leap: more than an ideology, almost a religion, inspired by a morbid fascination for the palingenetic power of the catastrophe.^5^ When this theory is applied to generative or evolutionary algorithms--when it stays within mathematics, and it applies to numbers--that's probably quite alright. That was John Holland's original idea.^6^ But when this theory is extended from technical systems to society and politics--then, I fear, we are all in deep trouble.

But this would be the topic for another paper.^7^ So let me just restate here briefly, where we stand now: The pandemic has proven that if we sympathize with the post-human biopolitics of the germs, and we let germs free to self-organize, germs will kill millions;^8^ just like recent political events have proven that if we sympathize with the post-modern identity politics of racist nation states, and we let the nationalists free to self-organize, they will kill millions, too. The pandemic--and for the last few weeks, war in Europe--have brutally reminded us that reality exists even without interpretations, and that there is a difference between fact and fiction, evidence and trial, reason and belief, science and voodoo--life and death. The pandemic and the war have proven that a complexist project for a self-organizing universe of emerging vitalities is a sure recipe for catastrophe; and they have proven that catastrophes, contrary to the principles of post-modern system theory, tend to be unpleasant for those who find themselves in the middle of them. The pandemic and the war are an unexpected and unwelcome revival of the real. Both remind us that non-human entities, such as objects, or germs, left to themselves, are not necessarily our best friends; and that the jungle, left to itself, is likely not the friendlier place to live. We do not need any more object-oriented ontologies these days; we need socially oriented computing; we urgently need a neo-modern, neo-rationalist and neo-digital agenda for a post-industrial world.

What the pandemic has proven, by temporarily but catastrophically obliterating every alternative, is this agenda exists, and it is viable. I just wish we should not have needed a global catastrophe to prove that we were right from the start. Back in the 90s we claimed that industrial modernity was doomed, and that post-industrial automation and computation were the only alternative. After 2001, that quickly became a lost cause--and often an unpopular one. Today, post-industrial computation is no longer a choice--it's our only remaining choice. There is no point in claiming that industrial modernity is unsustainable because industrial modernity is, literally, unsustained, having already imploded in full. There is no point in claiming that post-industrial computation is our future--because for the last two years post-industrial computation has been our present.

And as it happens, we, in the design professions, we have a few ideas on how we could make *post-industrial automation and computation* work. One generation ago we were among the first to conceptualize and theorize computational post-modernity; we were among the first to try to work with it, to try to make it work--first at small scale, then full-size. One generation later, we are still among the best in that trade. Today, all the digital and computational experiments we have been testing and trying for the last 30 years--from cyberspace to e-learning, from Virtual Reality and Augmented Reality to digital mass customization, micro-factories and distributed robotic automation--are primed and ready to go mainstream. In many ways, many of these computational tools have already gone mainstream--because for most of the last two years they have been indispensable; because during the COVID crisis we were forced to adopt them, and in the process, we learned that, if used advisedly, some computational tools for communication and for production and fabrication can actually work quite well. So let's try at least to learn something from the still ongoing catastrophe: this is not the time to relaunch a badly patched up version of the Anthropocene; this is the time to unbind a viable computational alternative to industrial modernity. And we know how to do it. We have been prepping for a long time.

## Footnotes

1 Mark C. Taylor and Esa Saarinen*, Imagologies. Media Philosophy* (London; New York, NY: Routledge, 1994).

2 Joseph Rykwert, *The Seduction of Place: The City in the Twenty-First Century* (New York, NY: Pantheon Books, 2000), 156.

3 The term "cyberspace" was invented by William Gibson, the noted science-fiction writer, in 1984. Gibson took his cue from Wiener's "cybernetics" (1948), a neologism Wiener derived from the Greek word for "steersman" (in Wiener's version, cybernetics originally referred to automatic piloting based on autocorrecting servomechanisms). In the 1990s cyberspace stood for any electronic alternative to physical space (hence most prominently the internet), but Gibson's original cyberspace, which was populated by electronic avatars, was closer to what today we would call the metaverse.

4 Published as “Coronavirus Might Give Us the Internet We Always Wanted,” *The Architect’s Newspaper*, New York, April 13, 2020, https://www.archpaper.com/2020/04/coronavirus-might-give-internet-weve-always-wanted/; reposted as “Postcoronial Studies: Coronavirus, and the Return of the Internet,” Labont Blog, Center for Ontology, Università di Torino, April 16, 2020, labontblog.com/2020/04/16/postcoronial-studies-coronavirus-and-the-return-of-the-internet/; and elsewhere with different titles. The expression “Post-coronial” is, to my knowledge, an invention of Professor Maurizio Ferraris (Center for Ontology of the University of Turin); see his best-selling *Post-Coronial Studies. Seicento Sfumature di Virus* (Turin: Einaudi, 2021). See also other opinion pieces I published in the *Architect's Newspaper* in the summer of 2020: "The On-line Tools Honed During the Pandemic Will Still Serve Us When It's Over," https://www.archpaper.com/2020/07/post-pandemic-potentials-the-online-tools-we-are-honing-will-still-serve-us/); "The Pandemic Changed Everything--Or So We Thought," https://www.archpaper.com/2020/06/the-pandemic-changed-everything-or-so-we-thought/)

5 On “complexism” as a “scientific ideology” see Christina Cogdell, *Towards a Living Architecture? Complexism and Biology in Generative Design* (Minneapolis, MN: University of Minnesota Press, 2018), 6, and passim*.*

6 John H. Holland, *Adaptation in Natural and Artificial Systems: an Introductory Analysis with Applications to Biology, Control, and Artificial Intelligence *(Ann Arbor: University of Michigan Press, 1975); “Genetic Algorithms: Computer programs that ‘evolve’ in ways that resemble natural selection can solve complex problems even their creators do not fully understand,” *Scientific American *267, no. 1 (July 1992): 66–73

7 It is in fact one of the subjects of my next book, *Beyond Digital: Design and Automation at the End of Modernity*, forthcoming with the MIT Press in 2023.

8 Benjamin Bratton, "18 Lessons of Quarantine Urbanism," *Strelka Magazine*, April 2020 (https://strelkamag.com/en/article/18-lessons-from-quarantine-urbanism); *Revenge of the Real. Politics for a Post-Pandemic World* (London: Verso, 2021).

